# Citrus/Cydonia Compositum Subcutaneous Injections versus Nasal Spray for Seasonal Allergic Rhinitis: A Randomized Controlled Trial on Efficacy and Safety

**DOI:** 10.5402/2011/836051

**Published:** 2011-07-18

**Authors:** Erik W. Baars, Miek Jong, Andreas F. M. Nierop, Inge Boers, Huub F. J. Savelkoul

**Affiliations:** ^1^Department of Healthcare and Nutrition, Louis Bolk Institute, Hoofdstraat 24, 3972 LA Driebergen, The Netherlands; ^2^Muvara, Tijmtuin 8, 2353 PH Leiderdorp, The Netherlands; ^3^Cell Biology and Immunology Group, Wageningen University, Marijkeweg 40, 6709 PG Wageningen, The Netherlands

## Abstract

*Background.* Clinical experiences *in vitro* and clinical studies have demonstrated the curative potency and safety of Citrus/Cydonia compositum in seasonal allergic rhinitis treatment. *Objectives.* To compare the efficacy and safety of two routes of administration (nasal spray versus subcutaneous injections). *Methodology: Design.* a national, randomised, comparative clinical trial with two parallel groups. *Participants.* 23 patients fulfilled the study requirements. *Intervention.* after a one- or two-week wash-out period, 23 patients were randomized, to a 6-week treatment period. *Outcomes.* immunological and symptom severity changes and safety. Immunologic outcome assessments were blinded to group assignment. 23 patients were randomized and from 22/23 patients (11 in each group) blood samples were analyzed before and after treatment. *Conclusion.* Both routes of administration demonstrate immunological and clinical effects, with larger inflammatory and innate immunological effects of the nasal spray route and larger allergen-specific clinical effects of the subcutaneous route, and are safe.

## 1. Introduction

Seasonal allergic rhinitis (SAR) or hay fever is a type I immediate hypersensitivity reaction mediated by specific IgE antibody formation to a seasonal allergen, leading to mucosal inflammation characterized by sneezing, itching, rhinorrhoea, and nasal blockage. Pollen from wind pollinated grasses, trees, weeds, and spores from fungi are the most common aeroallergens. The estimated prevalence of SAR in adults in several Western countries is 8–15% [1, 2]. The treatment of choice is the symptomatic treatment with antihistamines and/or local corticosteroids. Immunotherapy is indicated in a limited subpopulation of patients that are insufficiently treated with antihistamines and/or local corticosteroids [3]. Since SAR is a chronic disease and the treatment of choice for most patients is purely symptomatic, most SAR patients must be treated for decades. 

Citrus/Cydonia compositum (comp.) 1% solution for injection and Gencydo nasal spray are medicinal products, which contain exactly the same ratio of constituting substances, lemon juice (Citrus limon, succus), and an aqueous extract from quince (Cydonia oblonga, fructus rec.): 1 mL contains 8–12 mg Citrus limon, succus, corresponding to 0.65 mg fruit acid, calculated as citric acid and 30 mg aqueous extract from Cydonia oblonga, fructus rec. (1 : 2.1). For more than eighty years, Citrus/Cydonia comp. has been prescribed for SAR patients. 

The experiences of prescribing general practitioners (GPs) are that SAR patients are claiming to permanently suffer less from hay fever symptoms or even that they are free from complaints after the treatment with Citrus/Cydonia comp. [4]. Positive effects, without side effects, were also observed in two cohort studies: a group of 13 patients suffering from grass pollen SAR treated with subcutaneous injections [5] and a group of 140 patients, who were treated with nasal spray [6]. Recently, the immunological pathways underlying the positive effects of Citrus/Cydonia comp. in SAR patients were studied *in vitro* [7, 8]. These studies demonstrated a restoration of the disturbed immune state of allergic rhinitis patients by direct modulation of the Th1/Th2 balance. Such a perturbed Th1/Th2 balance is widely considered as the hallmark of allergic disease [9]. In addition, it was demonstrated that Citrus/Cydonia comp. significantly reduced the histamine production and the inflammatory mediator release from mast cells in a dose-dependent manner [8]. 

The objectives of this study were to assess and compare the immunological and clinical effects and to assess the safety of two routes of administration (subcutaneous injection versus nasal spray) of Citrus/Cydonia comp. 1% in order to determine which route of administration demonstrated superior efficacy and safety.

## 2. Methods

This is a national, stratified (age: 18–40 or 41–60, and RAST (radioallergosorbent testing) scores for birch pollen: >2 or <3, with a balanced randomization), comparative, and single-blind (laboratory) clinical trial with two parallel groups conducted in The Netherlands. 

### 2.1. Participants

Eligible participants were all adults aged 18 to 60, suffering from SAR for at least two years, with a RAST for grass pollen ≥2, suffering from the following nasal symptoms: sneezing, itching nose, and watery nasal discharge, with a severity score of at least two of the three symptoms ≥2 (ranging from 0 = not present to 3 = severe) and the necessity to use antihistamines and/or corticosteroids for treatment of symptoms for previous (at least two) years. Exclusion criteria were chronic inflammatory autoimmune diseases; allergic (hypersensitive) to one of the constituents of Citrus/Cydonia comp. or Gencydo nasal spray; pharmacological treatment of allergic rhinitis or use of other preparations containing Citrus and/or Cydonia extracts within the last two weeks prior to enrolment into the study; use of cromoglycates in the last month before study onset; concomitant pharmacological treatment indicated for seasonal allergic rhinitis such as antihistamines, corticosteroids, or other preparations; participation in a further clinical trial at the same time or within the previous 4 weeks prior to enrolment into this study; pregnancy or lactation; severe internal or systemic disease.

### 2.2. Ethics

The medical ethical committee (STEG-METC, Almere, The Netherlands) approved the study. Individual patients gave written informed consent.

### 2.3. Interventions

Patients received the treatment in accordance with the Summary of Product Characteristics; either Citrus/Cydonia comp. 1% subcutaneous injections (1 mL, ampoules available under the trade name Gencydo 1%, manufacturer Weleda AG, Schwäbisch Gmünd, Germany) twice per week, or the Gencydo nasal spray (1-2 sprays in each nostril) four times per day (available under the name “Gencydo neusspray” in the Netherlands, manufacturer Weleda AG, Schwäbisch Gmünd, Germany). This application strategy resulted in the nasal spray group receiving four times the active dose compared with the injection group. The composition of Citrus/Cydonia comp. 1% solution for injection and Gencydo nasal spray is identical. Both medicinal products contain lemon juice (*Citrus limon*) and an aqueous extract from the fruit of a quince (*Cydonia oblonga*): one milliliter of these preparations contains 8–12 mg *C. limon* juice corresponding to 0.65 mg fruit acid, calculated as citric acid, and 30 mg *C. oblonga* aqueous extract (drug-extraction-rate: 1 : 2.1).

### 2.4. Objectives

The objectives of the present study were to test (1) the immunological (primary objective) and clinical (secondary objective) superior efficacy of the subcutaneous route of administration compared to the nasal spray route of administration and (2) the safety of both routes of administration (tertiary objective) in a group of adult, grass pollen SAR patients. 

The primary hypothesis was that the subcutaneous route of administration demonstrated superior immunological efficacy, the secondary hypothesis was that the subcutaneous route of administration demonstrated superior clinical efficacy, and the third hypothesis was that both routes of administration were safe. Based on the results of the study, one route of administration will be studied in a future placebo-controlled, randomized trial.

### 2.5. Outcomes

Primary endpoints were SAR-related changes in immunological parameters between the start of the treatment (baseline) and after six weeks of treatment (postbaseline). From each patient 8 mL of peripheral blood was collected from which peripheral blood mononuclear cells (PBMCs) were isolated. PBMCs were cultured in Yssel's medium at 37°C in a humidified atmosphere with 5% CO_2_ at a density of 1 × 10^6^ viable cells/mL. Cells were plated out in 48 well plates at a concentration of 1 × 10^6^ cells/mL and cultured at 37°C. After five hours, in which the cells adapted to the culture conditions, various stimuli or a matching volume of medium was added. Cultures were stimulated polyclonally with 150 ng/mL anti-CD3 plus 100 ng/mL anti-CD28 monoclonal antibodies (BD Pharmingen, San Diego, Calif, USA) or cultured in medium only [10]. In addition, we performed allergen-specific stimulation of 10^6^ cells/ml in 1 mL cultures with applied pollen extract (Phl p 1 from Timothy grass, Phleum pretense; Biomay Vienna, Austria; 10 *μ*g/mL in medium).

 The proliferation capacity, cell survival, toxicity and total production capacity of several cytokines (e.g., IL-10, TNF-*α*, IFN-*γ*, IL-4, IL-5, and IL-13) in the culture supernatants of the PBMCs were analyzed at day 1 (demonstrating the reaction of the innate immune system) and day 7 (demonstrating the reactions of specialized T cells subsets) in the laboratory [10, 11]. The following changes in cytokine production levels were regarded as a positive immunological SAR treatment effect [12–14]:

1. the reduction of activation state of the SAR-related immune subsystem: reduction of (grass pollen stimulated minus medium stimulated) IL-10 and TNF-*α* at day 1;
2. the induction of (regulatory) T cells (Tregs): increase of (grass pollen stimulated minus medium stimulated) IL-10 at days 1 and 7;
3. the induction of Th1 activity: increase of (grass pollen stimulated minus medium stimulated) IFN-*γ* at days 1 and 7;
4. the reduction of Th2 activity: reduction of (grass pollen stimulated minus medium stimulated) IL-1*β* at day 1; IL-5 and IL-13 at day 7;
5. the reduction of chronic inflammatory activity: reduction of (grass pollen stimulated minus medium stimulated) TNF-*α* at day 1;
6. the restoration of the Th1/Th2 balance: the increase of (grass pollen stimulated minus medium stimulated) IFN-*γ*/IL-5 and IFN-*γ*/IL-13 ratios at day 7; and/or
7. the restoration of the Treg/Th2 balance: the increase of (grass pollen stimulated minus medium stimulated) IL-10/TNF-*α* ratio at day 1; the increase of (grass pollen stimulated minus medium stimulated) IL-10/IL-5 and IL-10/IL-13 ratios at day 7.

Secondary efficacy variables were the change in nasal and nonnasal allergic rhinitis symptom severity before treatment start and after each week of treatment. The severity of nasal symptoms (nasal obstruction, itching nose, sneezing, and watery nasal discharge) and nonnasal symptoms (itchy/burning eyes, watery eyes, redness of eyes, and itching ears/throat) symptoms were recorded twice per day (in the mornings and evenings) by the patient. The disease-specific symptom severity questionnaire was provided to the patient as an online questionnaire in Dutch: 0 = no symptom, 1 = mild, 2 = moderate, and 3 = severe. Completion of the online questionnaires by the participants was checked daily. 

Blood samples for immunological analyses were taken before and after six weeks of treatment. SAR related symptom severity scores were measured twice a day (morning and evening) during both the one-week or two-week washout and the six-week treatment periods.

Pollen counts were acquired on a daily basis for grass pollen and birch pollen from the Leiden University Medical Centre (http://www.lumc.nl/con/1070/85683/105795/105824/, Figure 2) during both the washout period and the treatment period. Safety was measured by means of adverse events surveillance and laboratory parameters (abnormal findings in the immunological analyses).

### 2.6. Sample Size

Based on an expected mean difference of IL-10 of 1.834 pg/mL, which is in line with the former *in vitro* studies [7], with a two-sided 5% significance level and a power of 95%, a sample size of 14 patients per group, thus in total 28 patients, was necessary.

### 2.7. Randomization-Sequence Generation

Prestratification on age (18–40 or 41–60) and RAST scores for grass pollen (>2 or <3) was used to divide participants into four subgroups. For allocation of the participants, a computer-generated list of random numbers was used. The randomization list was generated with the Random Allocation Software Program version 1.0 (Saghaei, Isfahan University of Medical Sciences, Iran) using a random block size of two in order to guarantee a balanced allocation.

### 2.8. Randomization-Allocation Concealment

After a one-week (for patients that had not been treated for SAR in the week before enrollment) or a two-week (for patients that had been treated for SAR in the week before enrollment) wash-out period, patients were assigned to four strata and then randomized to a six-week treatment period.

### 2.9. Randomization-Implementation

The two investigational medicinal products were assigned to treatment A and treatment B by the sponsor for all patients who were assigned randomly to either one of the two treatment groups. The label assignment was kept at the sponsor's site until all study data had been entered in the study database.

### 2.10. Blinding

The procedure of label assignment and random allocation to treatment A and treatment B was carried out to guarantee a blinded analysis of the primary efficacy variable, the immunological laboratory parameters. Laboratory personnel had no information about any treatment of patients during the period of all laboratory procedures and analyses. Unblinding took place after all statistics had been performed.

### 2.11. Statistical Analyses

Only evaluable patients, with immunological parameters measured before randomization and postbaseline (after six weeks of treatment) were included in the primary analysis for each primary efficacy variable (Per Protocol Set, PPS). In order to eliminate the impact of drop outs on efficacy results, in addition a subset of observed cases (OC) was evaluated. This subset included data only from randomized patients who did not discontinue prematurely and were available for evaluation at the designated assessment times. Missing values of the immunological parameters (e.g., values below cytokine detection limits) were not replaced. Missing values of the symptom severity scores, in absence of a major protocol violation, were replaced in two ways: by week means and last observation carried forward (LOCF) [15], and subsequently compared.

Regular descriptive statistics were performed with regard to demographical and categorical data. To test the primary hypothesis, superiority of Citrus/Cydonia comp. 1% solution for injection as compared to Gencydo nasal spray with respect to the primary target variable changes in immunological parameters, descriptive statistics, *Student's t-*tests, and nonparametric Wilcoxon Signed Ranks tests were performed to compare means and mean base-10 log transformed scores of the immunological parameters in both groups and to calculate 95% confidence intervals. 

To test the secondary hypothesis, superiority of Citrus/Cydonia comp. 1% solution for injection as compared to Gencydo nasal spray with respect to the secondary target variable lower seasonal allergic rhinitis symptom severity scores, mean scores and standard deviations per week were calculated. Then, multivariate analysis techniques were used to compare the symptom severity mean week scores in the subcutaneous and the nasal spray group for each of the 6 weeks and to calculate 95% confidence intervals. In addition, Cohen's delta was calculated for both routes of administration to estimate the effect sizes. 

The safety analysis was based on the Full Analysis Set (FAS) of all patients who took at least one dose of the randomised study medication. During the course of the study, all adverse events, irrespective of the relationship to the study medication or study procedure, were recorded on the adverse event forms contained in the Case Report Form (CRF). During each monitoring visit, the person responsible for monitoring of the clinical trial and the investigator reviewed all adverse events. With respect to all adverse events, the investigator was responsible for ensuring that correct and complete information was documented on the adverse event forms in the CRF. The assessment of the severity of an adverse event (AE) (mild, moderate, severe) was also performed by the investigator. The causal relationship with the administration of the investigational drug or a study procedure was assessed according to the categories as described by the Uppsala Monitoring Centre and recommended by the WHO (certain, probable, possible, unlikely, conditional, and unassessible), both by the investigator and the sponsor. 

## 3. Results

### 3.1. Participants Flow

**Figure 1:**
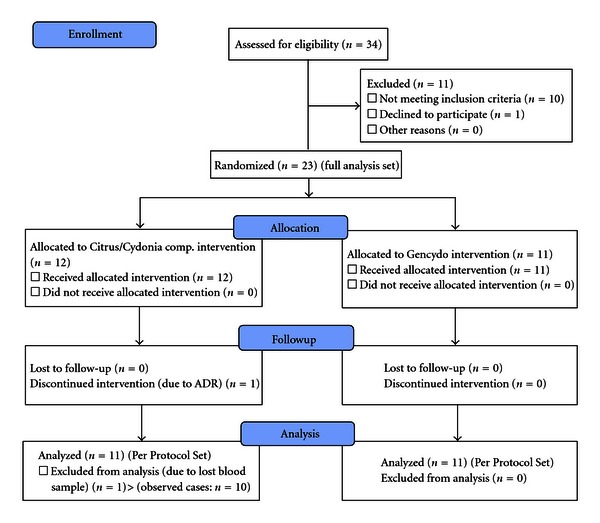
Participant flow: primary efficacy analysis data sets.

From 34 included patients, 11 patients dropped out before randomization either due to too low RAST score for grass pollen (*n* = 8), withdrawal of informed consent (*n* = 1), use of medication during wash-out period (*n* = 1) or too mild symptoms (*n* = 1). After randomization at baseline, the Full Analysis Set (FAS) contained 23 patients (12 patients in the Citrus/Cydonia group and 11 patients in the Gencydo group). There was one dropout in the Citrus/Cydonia group after three weeks of treatment due to an adverse event. From another patient in the Citrus/Cydonia group, the second blood sample got lost and therefore immunological analyses could not be performed. For the immunological analyses there were 22 patients (11 patients in each group) in the Per Protocol Set (PPS) and 21 patients (10 patients in the Citrus/Cydonia group and 11 patients in the Gencydo group) in the Observed Cases (OC) subgroup (Figure 1).

Patients were visited during the screening, before the start of the treatment and at the end of the treatment. A telephone visit was performed after three weeks of treatment. 

A total of 20 out of 23 patients (87%) started treatment in weeks 23 and 24: 10 of the 12 patients in the Citrus/Cydonia comp. group (83%) and 10 of the 11 patients in the Gencydo group (91%). Three other patients started in week 26: two patients in the Citrus/Cydonia comp. group and one patient in the Gencydo group (data not shown).

### 3.2. Recruitment

All eligible patients were recruited from a single centre, the Louis Bolk Institute (Driebergen, NL). The first patient was included on May 19, 2009 and the last patient completed the study on August 11, 2009.

### 3.3. Baseline Characteristics and Baseline Homogeneity

Baseline homogeneity of the treatment groups was accomplished with regard to the following SAR related aspects: RAST scores (grass pollen and birch pollen), worst SAR symptom severity during the previous pollen season (anamnestically), SAR symptom severity scores in the morning and the evening during the wash-out period (Table 1), and onset of interventions (data not shown). Homogeneity of the treatment groups was accomplished with regard to the following SAR nonrelated aspects: age, height, weight, smoking status, ethnic origin, remaining medical history, prior medication, vital signs, and physical examination. Homogeneity of the treatment groups was not accomplished with regard to gender as a SAR non-related aspect.

### 3.4. Pollen Counts

**Figure 2:**
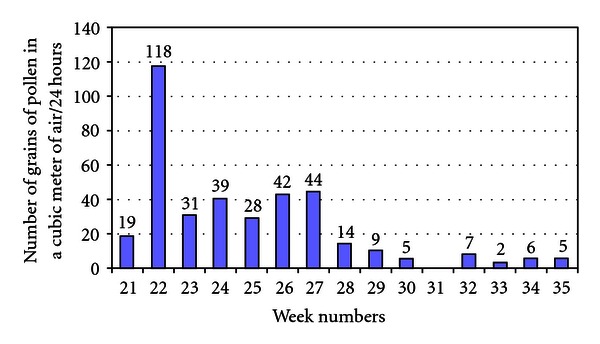
Mean grass pollen counts per week. Weeks 21–35 (2009).

The grass pollen counts during the wash-out period and the treatment period demonstrated that the grass pollen season in this period, apart from week 22, was not severe and the influence of the birch pollen was almost null (number of grains of pollen in a cubic meter of air/24 hours) (Figure 2). Grass pollen counts higher than 100 were measured in only four days in week 22, thus before the treatment period. After week 27, the week means were lower than 15. Since pollen counts higher than 80–100 are correlated with severe symptoms, and pollen counts higher than 10–15 are correlated with mild symptoms, the period after week 27 is not clinically relevant for this study [16].

Since patients had their wash-out period for one week or two weeks and in different weeks, the mean pollen count during the entire wash-out period was calculated. We calculated the exact mean pollen count in the wash-out period by adding all real pollen counts from day 1 to day 7 of the first wash-out week per patient and subsequently calculating the means of the pollen count. This resulted in a mean pollen count in the wash-out period of 44.6 (sd = 11.6) for the whole population, 46.8 (sd = 17.5) for the Citrus/Cydonia comp. group, and 39.1 (sd = 10.7) for the Gencydo group. These differences between both treatment groups were small, not statistically significant and clinically irrelevant, since the categories of symptom severity that are correlated to pollen count are mild (10/15–45/50), moderate (45/50–80/100), and severe (80/100 and higher), which implicated that both mean scores (39.1 and 46.8) were mild/(borderline) moderate.

### 3.5. Numbers Analyzed

23 patients were randomized (Full Analysis Set). From 22 patients (11 in each group) blood samples were analyzed before and after treatment (Per Protocol Set) and from 21 patients (10 patients in the Citrus/Cydonia group and 11 patients in the Gencydo group) in the Observed Cases (OC) subgroup (Figure 1).

From 20 of the 23 randomized patients (10 in each group), symptom severity scores were analyzed.

### 3.6. Primary Outcome Variables: Immunological Analyses

#### 3.6.1. Analyses at Day 1 of Allergen-Specific Stimulation

The analyses demonstrated acceptable cell survival, with no signs of toxicity (<5 % apoptotic cells, data not shown). Base-10 log transformations of the data, deemed mandatory due to an unequal distribution. 

The cytokine analyses of the PPS comparing medium stimulation versus allergen stimulation, demonstrated statistically significant increases both at baseline versus postbaseline of IL-10 (Gencydo group: 1.29 (95% CI: 1.15–1.44), *P* < .001 versus 0.97 (95% CI: 0.56–1.37), *P* < .001), (Citrus/Cydonia group: 1.03 (95% CI: 0.70–1.37), *P* < .001 versus 1.19 (95% CI: 0.64–1.74), *P* < .01) and TNF-*α* (Gencydo group: 1.44 (95% CI: 1.18–1.71), *P* < .001 versus 1.02 (95% CI: 0.51–1.53), *P* < .01) (Citrus/Cydonia group: 1.34 (95% CI: 0.76–1.91), *P* < .001 versus 0.96 (95% CI: 0.62–1.30, *P* < .001) in both treatment groups, but not of IFN-*γ* cytokine production (data not shown). 

Comparison of the results of allergen stimulation minus medium stimulation at baseline and postbaseline, demonstrated a reduction in TNF-*α* production level in the Gencydo group (−0.50 (95% CI: −0.08 to −0.93), *P* < .05), (Table 2). Comparing results of allergen stimulation at baseline and postbaseline demonstrated also a reduction of TNF-*α* in the Gencydo group (PPS: −0.36 (95% CI: −0.02 to −0.71), *P* < .05 versus OC: −0.33, *P* < .05). IL-10 and IFN-*γ* levels demonstrated no statistically significant changes. The analyses of the OC subgroup demonstrated no significant differences compared to the analyses of the PPS.

#### 3.6.2. Analyses at Day 7 of Allergen-Specific Stimulation

The analyses of the PPS and OC demonstrated only a baseline to postbaseline decrease of IL-10 cytokine production (Citrus/Cydonia comp. group (PPS versus OC): −0.68 (95% CI: −0.37 to −1.0), *P* < .01 versus −0.67, *P* < .05; Gencydo group: −0.44 (95% CI: −0.19 to −0.68), *P*<.01) and no statistically significant changes in all other cytokines (IL-12, IL-5, IL-13 and IFN-*γ* and relevant ratios of cytokines) in both groups (Table 3).

### 3.7. Secondary Efficacy Results: Symptom Severity

Total symptom scores (TSS) were analyzed during washout and week 1 until week 5 of treatment. Due to a very low pollen count during week 6, data of week 6 of treatment were excluded from the analyses. Missing values were replaced in two ways: mean week scores and Last Observation Carried Forward. When compared to the dataset without the missing values, the mean week scores demonstrate only small, non-significant differences (data not shown). 

**Figure 3:**
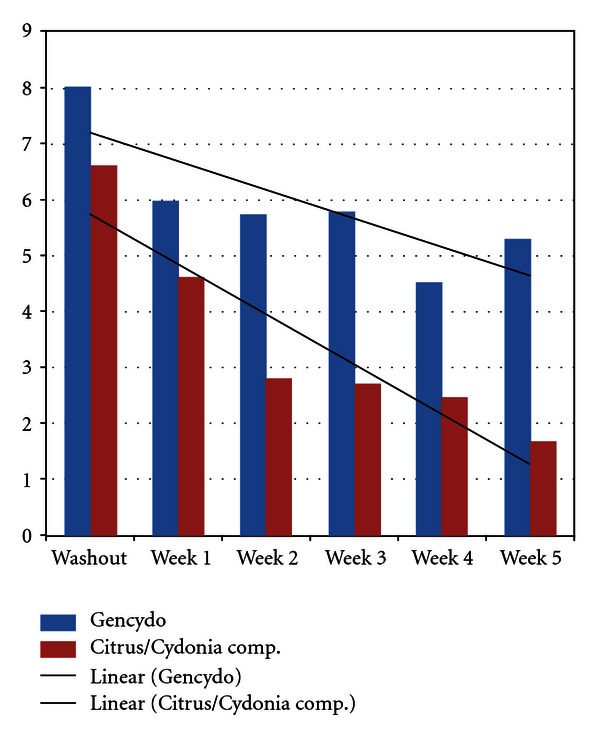
Mean total symptom scores from washout until five weeks of treatment in the *morning. *

A one-way-analysis of covariance (ANCOVA) with parallel regression lines of the TSS *morning* data demonstrated a statistically significant overall reduction of TSS scores in the period from washout to, respectively, 2, 3, 4, and 5 weeks (Figure 3). After three weeks of treatment until five weeks of treatment, there was also a statistically significant difference between (the level of the parallel regression lines of) the two treatment groups, demonstrating larger effects of the subcutaneous route of administration. Nonparametric tests demonstrated statistically significant differences between washout and all weeks of treatment (weeks 1–5) for the whole group, between washout and weeks 2–5 of treatment for the Citrus/Cydonia group, and no statistically significant differences between washout and all separate weeks of treatment for the Gencydo group. The TSS reduction in the Citrus/Cydonia group between washout and week 5 of treatment was 4.8 (95% C.I.: 1.7 to 7.9) (Table 4). The analyses of the PPS demonstrated small but not significant differences (data not shown).

**Figure 4:**
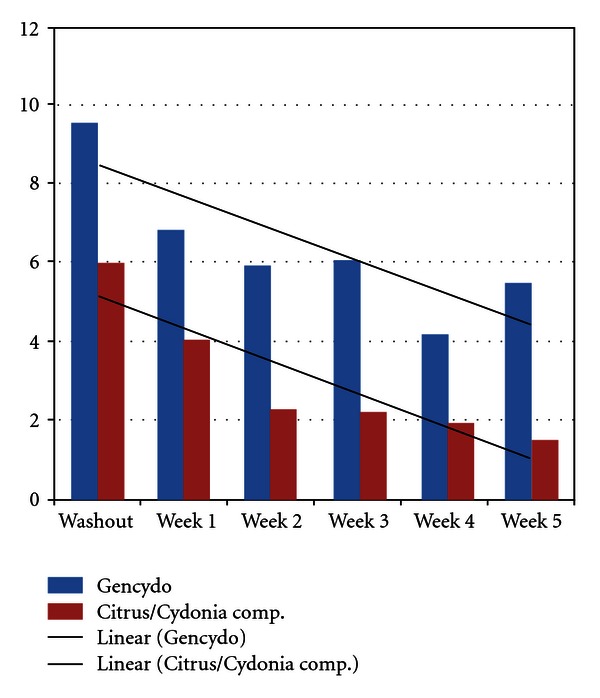
Mean total symptom scores from washout until five weeks of treatment in the *evening. *

A one-way-analysis of covariance (ANCOVA) with parallel regression lines of the TSS *evening* data demonstrates a statistically significant overall reduction of TSS scores in the period from washout to 2, 3, 4, and 5 weeks (Figure 4). There was a statistically significant difference between (the level of the parallel regression lines of) the two treatment groups at all times of treatment. Nonparametric tests demonstrated statistically significant differences between washout and all weeks of treatment (weeks 1–5) for the whole group, the Citrus/Cydonia group, and the Gencydo group. The TSS reduction between washout and week 5 of treatment was 4.5 (95% C.I.: 1.7 to 7.2) in the Citrus/Cydonia group, was 4.1 (95% C.I.: −0.4 to 7.8) in the Gencydo group, and was 4.3 (95% C.I.: 2.2 to 6.4) in the total group (Table 4). The analyses of the PPS demonstrated small but not significant differences (data not shown). 

Cohen's delta effect sizes (washout versus five weeks of treatment) were calculated for both the pooled data of all patients, and the Citrus/Cydonia group and the Gencydo group separately. Cohen's delta effect size for the pooled data of all patients in the morning was medium and large in the evening (resp.: 0.65 and 0.99). Cohen's delta effect sizes were large for the subcutaneous route of administration, both in the morning and the evening (resp.: 1.19 and 1.37), and for the nasal spray route of administration medium, both in the morning and the evening (resp.: 0.49 and 0.79) (but the nasal spray morning scores difference was not statistically significant). In order to control for possible bias due to the natural course of the disease (whereas the pollen count was very low after week 27), we post hoc reanalyzed the Cohen's delta effect sizes of all data without the data of the three patients that started treatment in week 26. Cohen's delta effect sizes for the pooled data of all patients now were large both in the morning and the evening (resp.: 0.98 and 0.81). Cohen's delta effect sizes again were large for the subcutaneous route of administration, both in the morning and the evening (resp.: 1.32 and 1.20), and for the nasal spray route of administration were now large in the morning and medium in the evening (respectively: 0.87 and 0.45) (but the nasal spray morning scores difference was still not statistically significant).

### 3.8. Adverse Events

The safety analysis set consisted of the 23 randomised patients of the Citrus/Cydonia and the Gencydo group. During the treatment period, a total of 9 adverse events (AEs) were observed in 6/23 patients (26.1%). The number of patients suffering from AEs in the Citrus/Cydonia group (five AEs in 2/12 patients; 16.7%), was lower compared to the Gencydo group (four AEs in 4/11 patients; 36.4%). The incidence of adverse events was comparable between the two treatment groups. None of the AEs were classified as serious. A causal relationship could not be excluded in all of these adverse events. In the Citrus/Cydonia group, three AEs were assessed as “probably related” and two as “possibly related.” In the Gencydo group, 3 AEs were assessed as “certain related” and 1 AE as “probably related.” One patient in the Citrus/Cydonia group terminated the study prematurely due to an AE (itching skin), which was assessed as “possibly related” to the study medication. Three of the nine AEs were of mild intensity, they all occurred in the Gencydo group. Two AEs in one patient in the Citrus/Cydonia group and one AE in the Gencydo group were classified as moderate. Three AEs in the Citrus/Cydonia group, all in one patient, were classified as severe. There were no serious AEs.

## 4. Discussion

In this study we compared two routes of administration nasal spray (Gencydo) versus subcutaneous injections (Citrus/Cydonia comp.) on immunological and clinical effects of SAR treatment and safety in a randomized controlled trial. The primary hypothesis was that the subcutaneous route of administration demonstrated superior immunological efficacy, the secondary hypothesis was that the subcutaneous route of administration demonstrated superior clinical efficacy, and the third hypothesis was that both routes of administration were safe. Based on the results of this study, one route of administration would be selected to be further tested in a placebo-controlled, randomized trial.

### 4.1. Interpretation

The immunological data comparing medium stimulation versus allergen stimulation reflect a rapid stimulation of the monocyte compartment in the stimulated PBMC fraction, and thus indicates the activation of a local innate immune response by the treatment in both treatment groups. In addition, the reduction of the production level of TNF-*α* in the Gencydo group reflects a decrease in the chronic SAR-related inflammatory activity between baseline and postbaseline. Overall, the observed kinetics at day 1, are consistent with a reduction of an allergic inflammatory condition with larger effects in the Gencydo group. This inhibition of inflammation is substantiated by the concomitant increase in monocyte-derived IL-10 production that profoundly suppresses the TNF-*α* production. The production level of monocyte-derived TNF-*α* and IL-10 reflects the local chronic SAR-related inflammatory activity between baseline and postbaseline. In addition, the observed kinetics at day 7 after allergen-specific stimulation, reflects the activation state of the immune system due to the activity of monocytes, which are the largest producers of IL-10 in the PBMC, and induced already by day 1 after allergen exposure. Subsequently, also the gradual and delayed induction of regulatory T-cell subset (Treg) by day 7 will be inhibited as these cells use the IL-10 as a selective autocrine growth factor [17, 18]. The observed kinetics can be interpreted as a decrease in the activation state of the immune system due to a decrease in the activity of monocytes, which are the largest producers of IL-10 in the PBMC, and induced already by day 1 after allergen exposure and a reduction of the chronic inflammation (TNF-*α* (day 1)). This additional effect can be attributed to the acute local effect of the nasal spray route. This decreased outgrowth of Tregs must then be the result of the effective treatment installed in these patients. The unaltered production levels of IL-5 and IL-13 (cytokines representing Th2-pathway) are consistent with a slower reacting T-cell compartment in these patients [19]. The frequency of allergen-specific T-cells will still be significant, probably in the order of 1 : 300, in these patients as they are in the pollen season and this arm of the immune system will still be triggered *in vivo* which compromises the Th2 analysis ex vivo [17, 20–22]. The monocyte compartment, being an essential part of the innate immune system, by definition will react faster on induced changes in the patient than the adaptive immune system, which is dependent on the frequency of allergen-specific T-cells and allergen-specific IgE antibody-forming B cells.

The overall conclusion on the immunological data is that both routes of administration demonstrate positive immunological effects on SAR-related cytokine production levels both in the innate reaction and in the reaction of the allergen-specific T cell subsets, with a larger innate reaction of the nasal spray route of administration. Both routes of administration appear to stimulate the monocyte compartment into a more immunoregulatory phenotype (day 1: medium stimulation compared to allergen stimulation increase of IL-10 and TNF-*α*). The day 1 results, reflecting the innate immune reaction, demonstrate more effect of the local Gencydo nasal spray route of administration with a reduction of the chronic inflammatory activity of the allergic Th2 pathway *in vivo* (baseline to postbaseline reduction of (allergen stimulation) TNF-*α*). The day 7 results demonstrate comparable effects in reduction of Treg production levels (baseline to postbaseline reduction of IL-10 (allergen stimulation)) of both routes of administration, which can be interpreted as a decrease in activation state of the immune system due to a decrease in the activity of both monocytes and regulatory T cells. 

The overall conclusion of the clinical data is that both routes of administration demonstrate a statistically significant reduction in SAR symptom severity, with larger effects of the subcutaneous route of administration. In the Citrus/Cydonia group a statistically significant SAR symptom reduction was measured already after one week and two weeks of treatment, in the morning and the evening, respectively. In the Gencydo group a statistically significant SAR symptom reduction was measured already after two weeks of treatment in the evening. TSS reduction of the subcutaneous route of administration was larger in the morning, but not in the evening. Cohen's delta effect sizes were larger for the subcutaneous route of administration than the nasal spray route of administration, both in the morning and the evening. During the treatment period, a total of 9 adverse events (AEs) were observed with none of the AEs classified as serious. Also the *in vitro* analyses demonstrated acceptable cell survival, with no signs of toxicity. The overall conclusion of the safety analysis in this study is that both routes of administration of Gencydo and Citrus/Cydonia comp. are safe for use by SAR patients.

This study demonstrates that both routes of administration have profound immunological and clinical effects on seasonal allergic rhinitis, with larger clinical effects of the subcutaneous route of administration and larger immunological (innate) effects of the nasal spray route of administration. Therefore, the primary hypothesis was rejected and the secondary hypothesis was confirmed. Both AEs analyses and *in vitro* immunological analyses demonstrate that both Citrus/Cydonia comp. and Gencydo are safe treatments, so that also the third hypothesis was confirmed. 

The small groups, the relatively low pollen counts during the study period and the absence of placebo groups for both the subcutaneous injection and the nasal spray routes of administration are the most important limitations of this study. The small groups might have led to an underestimation of both clinical and immunological differences between the treatment groups. Larger groups might have provided more precise estimations of means and smaller standard deviations, so that possible other immunological treatment effects might have been detected. The relatively low pollen counts have hampered to evaluate the efficacy of both routes of administration on severe SAR symptoms. However, the differential pollen counts during the short-term intervention during the allergen season did not influence the presence of peripheral blood T cells and their allergen-specific induced reaction profile. This is consistent with recent evidence showing that immunotherapy of birch pollen hay fever patients left allergen-specific Th2 cells unchanged after one year, but increased regulatory T cells that were considered responsible for the observed relief of symptoms. The expected rise in allergen-specific Th1 cells occurs even later after treatment [23]. The absence of placebo groups prevented the estimation of the exact specific effect of both routes of administration by means of controlling for placebo effects.

### 4.2. Generalizability

As the intervention was implemented for both sexes, adults from 18–60, both grass pollen and birch pollen SAR, the results indicate that a large subgroup of the SAR patient population might benefit from both routes of administration of this treatment.

### 4.3. Overall Evidence

The positive results of this study are in line with clinical experiences [4], *in vitro* studies [7, 8], and cohort studies [5, 6].

Based on the results of this study and previous studies, we can conclude that placebo-controlled clinical trials on short-term and long-term treatment are indicated and adequate to determine the specific effects of both routes of administration. Since the clinical effects (being the primary efficacy variable in SAR trials [24]) were larger in the subcutaneous route of administration and the day 7 immunological results were comparable, we choose to test the subcutaneous route of administration in future placebo-controlled clinical trials.

Since both the antihistamines and/or local corticosteroids treatment are purely symptomatic and immunotherapy is a treatment with (increasing doses of) pollen allergens, Citrus/Cydonia comp. must be regarded as a new, curative type of treatment that can potentially restore the disturbed immune state of SAR patients permanently. Since there is no stimulation of the immune system with gradually increasing doses of the substances to which a person is allergic, but more a controlled regulation of the activity of the immune system, another working mechanism regarding curative health promotion must be hypothesized.

## Figures and Tables

**Table 1:** Baseline Characteristics (Full Analysis Set).

Variable		Citrus/Cydonia comp.	Gencydo	*P* value
		(*n* = 12)	(*n* = 11)	
Sex: no. (percentage)	Male	7 (58%)	2 (18%)	.049*
	Female	5 (42%)	9 (82%)	
Age (year) (sd)		36.8 (12.4)	36.9 (10.7)	.97
Height (cm) (sd)		177 (9.5)	170.6 (7.4)	.09
Weight (kg) (sd)		70.8 (11.2)	66.4 (8.9)	.3
Smokers: no. (percentage)		3 (25%)	1 (9%)	.31
Alcohol consumption: no. (percentage)	None	0 (0%)	3 (27.3%)	.15
	Occasionally	11 (91.7%)	7 (63.6%)	
	Regularly	1 (8.3%)	1 (9%)	
Ethnic origin: no. (percentage)	Caucasian	12 (100%)	9 (82%)	.12
	Asian	0 (0%)	2 (18%)	
Childbearing potential:number (percentage)	Capable	3 (60%)	8 (88.9%)	.31
	Sterile	1 (20%)	1 (11.1%)	
	Postmenopausal	1 (20%)	0 (0%)	
Blood pressure at screening (mm Hg) (sd)		120 (23)/ 73 (14)	104 (16)/ 72 (9)	.09/.77
Heart rate at screening (beats per minute)		72 (9)	70 (6)	.56
RAST grass pollen		3.7 (1.2)	3.9 (1.2)	.76
RAST birch pollen		2.2 (1.8)	2.3 (2.1)	.82
Usual SAR symptom severity during the pollen season (total score anamnestically) (sd)	Sneezing	2.1 (0.3)	2.3 (0.5)	
	Itching nose	1.9 (0.7)	2.1 (0.5)	
	Watery nasal discharge	1.9 (0.7)	2.1 (0.5)	
	Total score	5.9 (1.4)	6.5 (1.2)	.5
SAR symptom severity scores in the morning during the wash-out period (total score) (sd)		6.6 (4.5)	8.0 (4.6)	.55
SAR symptom severity scores in the evening during the wash-out period (total score) (sd)		6.1 (3.7)	9.7 (5.2)	.19

*P < .05, **P < .01, ***P < .001.

**Table 2:** Log10 transformed allergen stimulation minus medium stimulation at day 1: baseline versus postbaseline cytokine production levels.

		Log10 (allergen stimulation minus medium stimulation) at baseline (range)	Log10 (allergen stimulation minus medium stimulation) at postbaseline (range)	Mean difference (95% CI)
Gencydo	IL-10^#^	1.91 (1.16–2.39)	1.85 (1.23–2.51)	ns
	IFN-*γ*	0.07 (0.01–0.34)	0.03 (0.1–0.28)	ns
	TNF-*α*	1.60 (1.16–2.12)	1.09 (0.25–2.08)	−.50* (−0.08 to −0.93)

Citrus/Cydonia	IL-10	1.64 (0.66–2.25)	1.60 (0.95–2.12)	ns
	IFN-*γ*	0.09 (0.1–0.50)	0.15 (0.1–0.84)	ns
	TNF-*α*	1.20 (−1.10–2.23)	1.34 (0.29–2.18)	ns

Total group	IL-10	1.77 (0.66–2.39)	1.73 (0.95–2.51)	ns
	IFN-*γ*	0.08 (0.1–0.50)	0.09 (0.1–0.84)	ns
	TNF-*α*	1.40 (−1.10–2.23)	1.21(0.25–2.18)	ns

*P value <.05.

**Table 3:** Changes in cytokine production at day 7 allergen-specific stimulation: baseline versus postbaseline.

Variable	Citrus/Cydonia comp.			Gencydo		
	Baseline (PPS/OC)	Postbaseline (PPS/OC)	Change (PPS/OC)(95% CI)	Baseline	Postbaseline	Change (95% CI)
IL-10^#^	2.36/2.37	1.68/1.70	−0.68*(−0.37 to −1.00)/−0.67*	2.22	1.79	−0.44*(−0.19 to −0.68)
IL-12	0.15/0.16	0.26/0.29	0.11/0.13	0.27	0.25	−0.02
IFN-*γ*	3.01/3.1	2.7/2.8	−0.31/−0.3	3.14	3.17	0.03
IL-5	2.21/2.27	2.06/2.05	−0.15/−0.22	2.29	2.3	0.01
IL-13	2.28/2.4	2.22/2.22	−0.06/−0.18	2.40	2.43	0.03

^#^All cytokine scores (IL-10, IL-12, IFN-*γ*, IL-5, and IL-13) are log10 transformed scores.

PPS = Per Protocol Set.

OC = Observed Cases.

*P < .05, **P < .01, ***P < .001.

**Table 4:** Mean total symptom scores washout versus five weeks of treatment: morning and evening scores (*n* = 20).

	Wash-out week mean (range)	Treatment week 5 mean (range)	Change (95% CI)
Morning scores			
Citrus/Cydonia comp.	6.6 (2.1–15.2)	1.7 (0–6.4)	4.8 (1.7–7.9)
Gencydo	8.0 (0.6–14.3)	5.3 (0.3–11.4)	ns
Total	7.3 (0.6–15.2)	3.5 (0–11.4)	3.8 (1.3–6.3)

Evening scores			
Citrus/Cydonia comp.	6.1 (1.7–11.8)	1.6 (0–5.0)	4.5 (1.7–7.2)
Gencydo	9.7 (0.3–16.5)	5.6 (0–15.0)	4.1 (0.4–7.8)
Total	8.0 (0.3–16.5)	3.6 (0–15.0)	4.3 (2.2–6.4)

Total symptom scores can vary from 0–24: 0–8: mild; 9–16: moderate; 17–24: severe.
